# Salol in the Treatment of Cystitis

**Published:** 1889-03

**Authors:** E. L. Vansant

**Affiliations:** Demonstrator of Pathological Histology; Chief assistant, Medical Clinic, Medico-Chirurgical College


### SALOL IN THE TREATMENT OF CYSTITIS.

By. E. L. VANSANT, M. D.,

Demoustrator of Pathological Histology; Chief assistant, Medical Clinic, Medico-Chirurgical College.

The results in the treatment of catarrhal cystitis by salol have been so satisfactory that it seems proper to draw further attention to its use in this affection.

I have used it now in a number of cases and the results in each case have been surprisingly rapid and beneficial.

The following three cases have been selected as good illustrations :

Case 1. John M., aged 65.

Jan. 7, 1889. Complains of pain in the lumbar region; frequent micturation, accompanied by straining and pain; passes his urine every three hours by day and arises three or four times at night; appetite is poor; has dizziness and indigestion. Examination of the urine: pale, cloudy, with considerable sediment; reaction slightly acid; sp. gr. 1022; tests for albumen and sugar negative. Microscopical examination of the urine gives epithelial cells and pus corpuscles. Examination of the prostate shows right lobe somewhat enlarged; left lobe seems normal; the entire gland sensitive to the touch. Examination of the bladder per catheter shows that the patient is capable of emptying that viscus.

Diagnosis: chronic catarrhal cystitis.

Treatment: salol, gr. v, every four hours.

Jan. 10. Reports feeling very much better. Now passes urine three times during the day and twice at night; urination is unaccompanied by pain or straining; pain in the back much less, appetite good, bowels regular. Examination of the urine : clear amber color, no sediment, reaction strongly acid, sp. gr. 1022, no albumen, etc. Treatment discontinued.

Jan. 14. Remains in the same improved condition as at last visit.

Case 2. Margaret M., aged 60, patient has been under treatment for some time for phthisis pulmonalis.

Jan. 12, 1889. Returns complaining of having caught cold about two weeks ago, and now suffers from frequent urination accompanied by severe straining and burning. She says that the desire to urinate is almost constant, and that she arises frequently at night; also has severe pain in the back. These symptoms are of about two weeks, duration. Examination of the urine: pale, cloudy, with considerable sediment; reaction slightly acid, sp. gr. 1020, slight amount of albumen. Microscopical examination gives epithelial cells from bladder and vagina, pus corpuscles and some red blood corpuscles.

Diagnosis: subacute catarrhal cystitis. Treatment: salol, gr. v, every four hours.

Jan. 14. Reports that the painful micturition ceased yesterday afternoon and has had no pain since. She arose once last night and has urinated twice to-day before 8 p. m. Examination of the urine: amber color, very slight sediment, reaction strongly acid, sp. gr. 1020, no albumen. Microscopical examination: some epithelial cells and pus corpuscles.

Jan. 17. Condition continues good.

Case 3. Dr.——complains of slight catarrhal cystitis with frequent micturition, for past several months. Urine not examined. Treatment: salol, gr. v, every four hours. Reports, two days later, entire relief, which continues up to the present time.

The mode of administration followed has been the same in each case: the remedy was ordered in pill form and given in five-grain doses every four hours.

The results from this quantity were so satisfactory that other sized doses have not as yet been tried. Whether one single large dose will not perform the same work is, I think, worthy of investigation.—*Med. Times.*

